# How to Define the Latent Reservoir: Tools of the Trade

**DOI:** 10.1007/s11904-016-0304-1

**Published:** 2016-02-11

**Authors:** Kirston M. Barton, Sarah E. Palmer

**Affiliations:** Westmead Millennium Institute/University of Sydney, 176 Hawkesbury Road, Westmead, NSW 2145 Australia

**Keywords:** Human immunodeficiency virus, Persistent HIV, Latent HIV, Viral outgrowth assay, Single-copy assay, HIV biomarkers, HIV pathogenesis, Review

## Abstract

HIV is a devastating worldwide epidemic that has had substantial social and economic impacts throughout the globe. Due to the presence of a small pool of latently infected cells that persists during antiretroviral therapy (ART), HIV is not curable. Because of the high cost of ART and the lack of reliable accessibility across the globe, life-long ART is unfortunately not a feasible solution for the epidemic. Therefore, new strategies need to be developed and implemented to address HIV-1 infection. Several approaches toward this end are currently under investigation (Ebina et al. in Sci Rep 3:2510, 2013; Archin et al. in Nature 487:482–5, 2012; Elliott et al. in PLoS Pathog 10:e1004473, 2014; Rasmussen et al. in Lancet HIV 1:e13–e21, 2014; Tebas et al. in N Engl J Med 370:901–10, 2014; Archin et al. in Nat Rev Microbiol 12:750–64, 2014; Barton et al. in PLoS One 9:e102684, 2014; Sogaard et al. in PLoS Pathog 11:e1005142, 2015). Initial studies have proven promising, but have highlighted the need for sensitive and accurate assays to detect changes in very low concentrations of virus to allow confident interpretation of the success of curative approaches. This review will focus on assays that are currently available and the advantages and limitations of each.

## Introduction

Antiretroviral therapy (ART) effectively reduces the amount of HIV-1 in infected individuals to levels that are undetectable by most clinical measures. However, the small reservoir of virus that is able to persist has the capacity to develop into a full HIV-1 infection if ART is stopped [1]. Therefore, this reservoir has important implications for infected individuals [2]. To cure HIV-1, gene therapy, bone marrow transplant, and combined pharmacological and immune therapy are some of the most well-known methods that are being actively explored. An accurate and sensitive measure of the latent reservoir is needed to measure outcomes of these studies [3, 4, 5••,^6^••,^7^--^9^,^10^••]. The persistent reservoir of latent HIV-1 that remains during effective long-term therapy has been intensely studied, and several important features of the reservoir have been elucidated. The most well-studied reservoir exists in the resting CD4-positive T cells of the peripheral blood and in the intestine [11, 12]. Most measures of the persistent pool of HIV-1 focus on the peripheral blood as a surrogate marker of other reservoirs due to its accessibility. However, alternate reservoirs of persistent HIV-1 have been detected in the lymph nodes, seminal fluid, and cerebral spinal fluid [11, 13]. The majority of infected cells contain only a single HIV-1 integrant, meaning that measurement of integrated cell-associated DNA generally correlates with the number of cells that are infected with HIV [14, 15••]. Once ART therapy is initiated, no viral evolution is detected indicating that there is no ongoing viral replication when ART adherence is high [16]. However, while HIV-1 does not replicate, the pool of latently infected cells can maintain or expand through cell proliferation of infected cells [17]. Therefore, while the persistent reservoir is not expanded through ongoing HIV-1 replication, due to the presence of long-lived cells and cell proliferation, it can be maintained over time. These important studies have paved the way for future assays to measure the latent reservoir and allow informed evaluation of the assays in use. To date, several innovative assays that measure various aspects of the latent reservoir have been developed, and these assays are being used to direct clinical decisions regarding continuation of ART.

To fully understand the difficulty of developing an effective and efficient measure of the latent reservoir, it is important to define what an ideal assay would include. The first and most important feature, but possibly the most difficult, would be to measure the entire reservoir of replication competent virus and nothing more. Specifically, the assay would need to quantify replication competent HIV-1 without including defective virus. The majority of HIV-1 proviral genomes that persist in HIV-1-infected individuals on long-term suppressive therapy are defective in that they contain premature stop codons and/or large internal deletions and insertions [18•]. It is important to distinguish between defective and replication competent virus because in theory, HIV-1-infected individuals could remain off ART indefinitely if all replication competent viruses are eliminated while cells containing defective virus remain. However, defective virus that is not replication competent, but makes partial viral proteins, can cause persistent immune activation. Persistent immune activation also has long-term deleterious effects, and therefore, sterilizing strategies that remove all HIV-1, such as gene therapy, may ultimately prove to be an optimal therapeutic choice.

Second, an ideal assay would be able to provide an accurate assessment of how much replication competent virus is present in tissue compartments that are not necessarily sampled. Due to the limited amount of interventions a participant can reasonably be expected to endure to efficiently deploy an assay across the many health-care systems around the globe, an ideal assay would require only a single blood sample, but would need to be able to accurately predict the levels of HIV-1 in tissue compartments such as the intestine, the lymph nodes, the nervous system, and yet undefined populations. Third, an ideal assay would have very strong confidence in a negative result as false negatives would have devastating effects on the patient. While this may seem like a difficult task at first glance, combining current methods with theoretical modeling may provide sufficient data to make initial predictions while more sensitive and deployable methods are developed.

## Assays to Measure the Persistent Reservoir

The first group of assays that is going to be discussed measures virus that is replication competent and can infect cells in culture. Collectively, these assays use viral outgrowth to measure the latent reservoir. So far, three assays have been developed. As a group, viral outgrowth assays are very promising because they are the only class of assays that detect only replication competent virus. However, they share a common limitation in that they are not necessarily able to detect all replication competent viruses and, if used as the sole predictor of reservoir size could, lead to premature discontinuation of ART [18•].

The quantitative viral outgrowth assay (QVOA) is currently the gold standard for detection of the HIV-1 latent reservoir [1, 19, 20]. In this assay, blood or leukopheresis product is obtained from HIV-1-infected individuals, and CD4+ T cells are isolated by negative selection. The CD4+ T cells are plated in limiting dilution to allow quantification and are stimulated with PHA and irradiated lymphoblasts from a non-infected donor to induce viral outgrowth. Then, CD8+ depleted lymphoblasts are added to amplify the virus that is released. After several weeks of incubation, the wells that contained replication competent HIV-1 are detected by ELISA for HIV-1 p24 antigen. QVOA has many advantages with the most pronounced being its ability to only detect replication competent virus. However, it is also costly, time-consuming, and resource-intensive; requires large samples from the study participants; and does not detect all replication competent virus [18•, 21]. Several variants of this assay have been developed to reduce the cost and length of QVOA including the use of MOLT-4/CCR5 cells in place of donor cells for viral expansion, which decreases the amount of required culture time [22]. This assay also reduces the total time by detecting HIV-1 RNA with quantitative PCR (qPCR) instead of using ELISA. The initial steps of the viral outgrowth assay are followed; however, culturing is stopped after only three or seven days, and viral RNA is detected by qPCR [22].

An alternative to the traditional QVOA was recently proposed by Swanstrom et al. in which the maximum number of CD4+ T cells (ten million) is cultured in each well, and then, the positive wells are sequenced using ultra-deep sequencing to determine the number of unique viruses that replicated in each well. This approach allows more cells to be cultured with fewer resources. One minor limitation to this assay is that it is less accurate in participants treated during acute infection due to a lack of genetic diversity, which is necessary to be able to quantitate the number of individual founder viruses were present in each well [23].

Recently, another variant of the viral outgrowth assay using humanized mice (MVOA) was developed. In this assay, CD4^+^ T cells or PBMCs from HIV-infected individuals are injected into Nod.CgPrkdc^scid^Il2rg^Tm1Wj1^/SzJ (NSG) mice. Viral loads are then monitored using real-time PCR for HIV1 RNA. Up to 60 million cells can be injected per mouse, and multiple mice can be used per HIV-1-infected individual increasing the number of cells that can be screened. In the trials that have been run so far, HIV-1 was detected in mice injected with cells from participants on ART and elite controllers with viral loads less than 50 copies per milliliter. HIV-1 RNA was detected in the plasma of the humanized mice after xenograft with PBMCs from five participants who were on effective long-term ART and from six elite controllers. MVOA also has the potential to screen cells from alternate tissue reservoirs such as the gut-associated lymphoid tissue and lymph nodes. This assay has a higher sensitivity than the QVOA but is not quantitative and is thus limited to determining whether the participant still harbors replication competent virus [24]. MVOA is a very powerful tool for determining whether replication competent virus is present in large amounts of cells. However, the MVOA assay would be less informative in detecting fold reductions in the latent reservoir that could potentially lead to long periods of HIV-1 remission.

Residual viremia can be detected in a significant portion of HIV-1-infected individuals on effective therapy using very sensitive methods [25, 26]. The single-copy assay (SCA) is a sensitive real-time RT-PCR-based assay that can measure down to one copy of viral RNA per milliliter of plasma [25–29]. SCA uses primers directed at the highly conserved regions of *gag* and *integrase*. Comparison to serial dilutions of a standard is used to quantify the total amount of viral RNA. In addition, an internal standard (avian retrovirus) is added to all plasma samples to monitor the extraction and measurement of low-level viral RNA. SCA is more sensitive than any of the clinical detection methods that are currently available. Commercial assays including the Roche Ampliprep [30] and Abbot real time HIV-1 assays can also be used to measure HIV-1 RNA in the plasma. These assays are much less sensitive than the SCA with lower limits of 20 or 40 copies per milliliter, respectively. Assays that measure plasma RNA do not detect latent virus that is not producing transcripts and do not determine whether the virus is replication competent. Furthermore, because they use specific primers, some proportion of virus may not be detected due to mutation or viral variation. Despite these limitations, the single-copy assay can often detect plasma RNA in individuals on ART.

During the HIV-1 replication cycle, several nucleic acid forms of the viral genome are created including full-length viral RNA, 2-LTR DNA circles, integrated DNA, as well as multiply spliced and incompletely spliced mRNA. Quantitative PCR assays can be used to distinguish between each form or to measure total RNA or DNA according to the amplification region [31]. It is important to carefully select which region is targeted by these assays. Short abortive transcripts from the HIV-1 LTR can be detected in the absence of full transcription due to limitations of the elongation machinery [32]. Additionally, regions such as *env* are known to be highly diverse, which makes false negatives due to primer mismatch more likely. Protein regions such as *tat* and *integrase* are highly conserved and are therefore less likely to produce false negative results. When measuring RNA, it is important to keep in mind that fully spliced RNAs are produced early in infection and encode *rev*, which is required for nuclear export and translation of multiply spliced and unspliced HIV-1 RNA. Furthermore, HIV-1 integrated into an exon of a gene can be translated as a full-length genome within that gene without the potential to be translated into functional proteins or be incorporated into a virion as a viral genome [2, 11]. Additionally, integrated DNA species that contain mutations rendering them defective may still produce RNAs. Defective integrated HIV-1 genomes can be propagated through cellular proliferation due to antigenic or homeostatic proliferation. Multiply spliced RNAs are highly expressed during early infection peaking at five hours while unspliced RNAs dominate at later stages of infection [33]. Furthermore, multi-spliced RNAs are generally undetectable in individuals on ART while unspliced RNA can be detected at a very low level [32]. A ratio of the two species can be used to provide an idea of whether new infections are occurring [32, 34, 35]. Therefore, detection of each type of viral RNA or DNA provides an independent measure with varying amounts of reliability regarding its ability to predict how much replication competent HIV-1 is present [18•, 36].

The PCR-based assays are the most flexible group of assays available in that they are able to differentiate between many different stages of the viral life cycle [11, 37–41]. Additionally, these assays are very sensitive and specific. Most assays in use rely on quantitative real-time PCR, while some of the newer methods have moved to digital droplet PCR (ddPCR) to increase sensitivity [42]. Measurement of total DNA includes both integrated and 2-LTR circle forms of the HIV-1 genome [43, 44]. While the longevity of 2-LTR circles is controversial, accurate measures of integrated HIV-1 genomes can be measured using *Alu* PCR [29, 45–47]. *Alu* PCR is a method that takes advantage of very common *Alu* elements that are present in the human genome along with HIV-1-specific primers that bind to the *gag* region. Together, these primer sets are used to detect integrated HIV-1 DNA in cells [48–50].

The tat/rev induced limiting dilution assay (TILDA) measures inducible multiply spliced HIV-1 RNA [51]. This approach is a better predictor of the actual reservoir size than other PCR-based methods because it measures RNAs produced in response to stimulation with PMA indicating that the proviral LTR is intact. Furthermore, the primers used for TILDA are specific to the tat/rev region, which is the most commonly deleted region in proviruses. In one study, TILDA predicted a latent reservoir that was 48 times higher than that predicted by QVOA and 6–27 times lower than that predicted by PCR-based assays, supporting its ability to measure a larger proportion of the latent reservoir than QVOA while being more discriminating than traditional PCR-based approaches [51].

While highly sensitive, none of the PCR-based assays are able to fully differentiate between replication competent and defective virus, meaning that for the most part, they may over estimate the total latent reservoir. Additionally, due to the capricious nature of the HIV-1 genome, even primers targeting the most conserved regions may miss some sequences. Moreover, many of the primers used for PCR assays are somewhat subtype-specific, and therefore, knowledge of the subtype may be required for optimal implementation. Despite these drawbacks, PCR-based assays are some of the least expensive and most transferable assays available. Furthermore, PCR assays may be able to be combined with sequencing data to estimate the percent of detected HIV that is defective and provide a more accurate measure of the latent reservoir.

Measures of the immune response can also be used as biomarkers to predict HIV-1-free remission. Several different measures of immune activity are available including HIV-1 antibody detection, response of T cells to HIV-1 antigen, and markers of activated T cells. To measure HIV-1-specific antibodies, the most common method in use is ELISA. ELISA can detect both the avidity and quantity of HIV-1 antibodies and can be used to predict whether the participant is acutely or chronically infected with HIV [52]. Two recent studies following participants with stem cell transplants both showed declines in HIV-1 antibodies over time [53••, 54••]. However, this decline is associated with a continued remission for one individual while it was only associated with a delay in viral rebound for the other two individuals. Therefore, while the levels and avidity of HIV-1 antibodies can be used as a predictor of HIV-1 levels, this method may need to be supplemented with additional measures. The luciferase immunoprecipitation system, which uses chimeric *Renilla* luciferase and pathogen-specific antigens to quantitate HIV-1-specific antibodies, demonstrated that the levels of antibodies to p24 and gp41 were significantly lower in a participant that has not rebounded than in elite controllers who control viremia without ART and HIV-infected individuals on or not on ART. Therefore, the levels of p24- and gp41-specific antibodies may be a surrogate biomarker of reservoir size [55].

HIV-specific T cell responses can also be used as a measure of recent exposure of the immune system to viral proteins. In this assay, total PBMCs are exposed to HIV-1 peptides and intramolecular staining for known cytokines is used to determine whether CD4 and CD8 T cells are responsive to HIV. A decline of T cell specificity for HIV-1 peptides indicates that the immune system has not been exposed to antigen recently. However, a recent study by Oleson et al. found that in their study group, cytokine expression was not correlated with levels of HIV DNA; however, they did find that the frequency of natural killer cells was inversely correlated with levels of HIV DNA in infected individuals [56]. In addition to the response of T cells, cell surface proteins that correlate with cell activation are biomarkers that may be useful to predict reservoir size. Levels of HIV-1 proviral DNA are significantly correlated with markers of cell activation with the strongest association being observed between HIV-1 proviral DNA and the markers PD-1 and HLA-DR [57, 58]. Therefore, PD-1 and HLA-DR expression could also potentially be used as a alternate markers for the presence of residual HIV-1.

One common feature of all the assays that have been discussed above is that they all are largely used and optimized in peripheral blood samples. However, the lymph node and gut-associated lymph node tissues are also well-known reservoirs of persistent HIV. As stated above, an ideal assay would use a small blood sample and provide a prediction of the total pool of persistent HIV. However, to determine whether an assay is a good predictor of the total reservoir, it is important to have a thorough understanding of how much persistent HIV is in each tissue reservoir. RNA/DNAscope (NAscope) was developed by Jacob Estes to detect HIV-1 RNA and DNA in tissue specimens [59]. NAScope is a modified version of traditional in-situ hybridization that is more specific allowing individually infected cells to be detected and quantitated. The PCR-based methods discussed above as well as traditional in-situ hybridization techniques have also been used to detect HIV-1 in tissue samples [54••, 60–63]. Sequencing can also be used to characterize persistent reservoirs in tissue samples and compare them to the peripheral blood components. Sequencing studies of the lymph nodes and gut-associated lymph node tissues have largely showed that these populations intermingle with HIV populations from the peripheral blood indicating that HIV-1 regularly traffics between these compartments [16]. Analysis of the cerebral spinal fluid in some individuals does indicate that compartmentalization can occur across the blood brain barrier [64]. As new curative strategies progress from the initial phases, it will be crucial that the reservoirs in the tissue compartment be quantifiable so that the effects of new strategies can be measured.

ART treatment interruption is the ultimate measure of whether replication competent virus persists. Many have reservations about using treatment interruption as lack of ART adherence and treatment interruptions has been shown to correlate with poorer health outcomes for the patients [65, 66]. However, these studies were not designed to closely monitor participants and place them back on ART as is proposed for the analytical treatment interruptions to test the efficacy of curative/remission strategies. Additional studies need to be conducted to determine the full effects of well-controlled analytical treatment interruptions and whether the participants are significantly negatively affected.

A functional cure in which all replication competent virus is eliminated is the ultimate goal of HIV-1 research. However, recent studies have indicated that decreasing the HIV-1 reservoir by several folds may be sufficient to effect HIV-1 remission during which HIV-1 infected individuals could stop taking therapy for months or years without recurrent viremia. The Davenport lab analyzed the results from four cohorts and a total of 100 participants who underwent treatment interruption and determined that on average, a new virus reactivates every five to eight days [67•]. Furthermore, based on this number, they estimate that a 50–70-fold reduction in the viral reservoir would be needed to achieve HIV-1 remission for approximately one year, which is less than the 2000-fold reduction that was previously estimated [67•, 68]. Most of the assays that are currently being used to estimate the latent reservoir are close to the limit of detection with the current levels of persistent HIV-1 in participants on long-term effective therapy. To be able to detect a 50–70-fold reduction in these levels, significantly more sensitive assays and technologies will need to be developed.

**Fig. 1:**
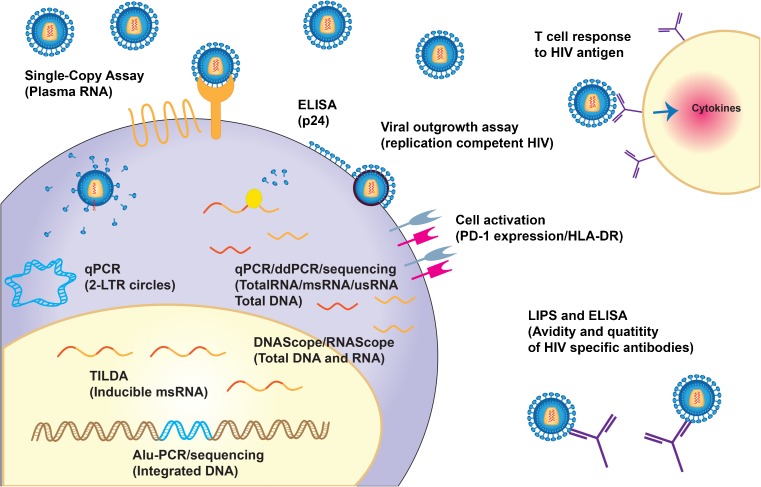
The many different stages of the HIV replication cycle and the response of the host’s immune system can be detected with sensitive assays to quantitate HIV in HIV-infected individuals on effective long-term antiretroviral therapy

Eriksson et al. compared some of the most common assays that are used to measure the latent reservoir including qPCR and ddPCR for HIV-1 DNA, *Alu* PCR for integrated HIV-1 DNA, DNA and RNA in the gut-associated lymphoid tissue, ddPCR for 2-LTR circles, and the single-copy assay, and compared them to results from the same study participants using the viral outgrowth assay [69••]. None of the measurements from the techniques studied were strongly correlated with the results from the viral outgrowth assay, which is not surprising given that each assay measures very different aspects of HIV-1 replication that may expand or contract independently (Fig. 1). Additionally, Chun et al. compared HIV-1 DNA to plasma viremia and markers of immune activation and found a correlation between plasma viremia and cell-associated DNA, but did not observe a significant correlation when they compared cell-associated DNA to markers of immune activation [41]. These studies highlight the fact that with the currently available technologies, the results from multiple methods may need to be combined to predict a confident estimate of the size of the reservoir.

In addition to these studies, a large collaboration between several different labs using most of the current techniques available with a large range of anatomical samples attempted to detect HIV-1 in the longest case of HIV-1 remission currently known as the Berlin patient [54••]. HIV-1 RNA/DNA was detected at very low levels in three independent labs; however, to date, no viral rebound has occurred. In contrast, a similar study was performed on two participants who received bone marrow transplants in which no virus was detected by any of the methods used. However, both of these individuals experienced rebound following treatment interruption [53••]. These two studies highlight the difficulty of accurately measuring the latent reservoir.

## Conclusions

In conclusion, many innovative and sensitive assays are available to measure and predict the size of the persistent reservoir in HIV-1-infected individuals on long-term suppressive therapy. Despite some limitations for each assay, combining these techniques may provide a powerful estimation of the latent reservoir. A clear understanding of each assay and how to interpret the information provided is critical for translating the findings into meaningful recommendations for patients. Even with the best and most careful measurements, it is likely that well-monitored analytical treatment interruptions will provide the most significant insights into the effectiveness of cure and remission studies.
